# Building a Rechargeable Voltaic Battery via Reversible Oxide Anion Insertion in Copper Electrodes

**DOI:** 10.1021/acsaem.4c00008

**Published:** 2024-02-23

**Authors:** Jose Fernando Florez Gomez, Nischal Oli, Songyang Chang, Shen Qiu, Swati Katiyar, Ram Katiyar, Gerardo Morell, Xianyong Wu

**Affiliations:** † Department of Physics, University of Puerto Rico-Rio Piedras Campus, San Juan, Puerto Rico 00925-2537, United States; ‡ Department of Chemistry, University of Puerto Rico-Rio Piedras Campus, San Juan, Puerto Rico 00925-2537, United States

**Keywords:** rechargeable voltaic batteries, zinc metal batteries, anion insertion, nanosized copper, aqueous electrolytes

## Abstract

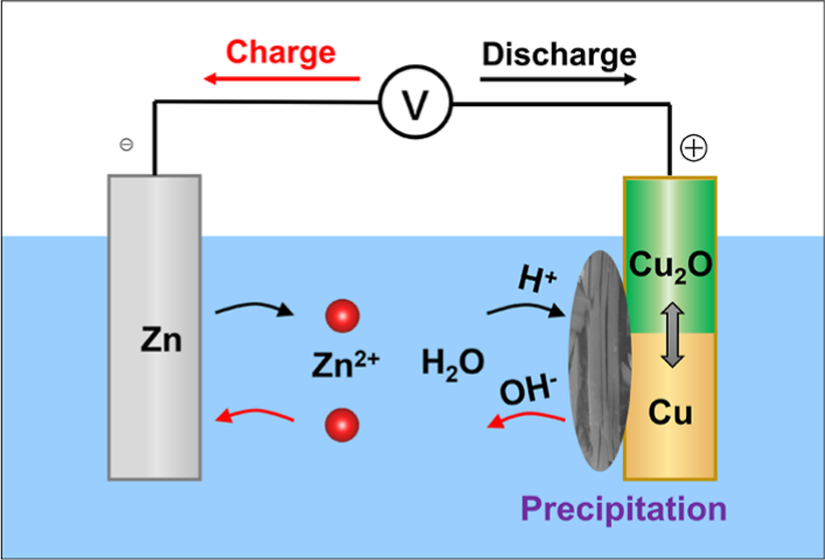

Voltaic pile, the very first battery built by humanity in 1800, plays a seminal role in battery development history. However, the premature design leads to the inevitable copper ion dissolution issue, which dictates its primary battery nature. To address this issue, solid-state electrolytes, ion exchange membranes, and/or sophisticated electrolytes are widely utilized, leading to high costs and complicated cell configuration. Herein, we build a rechargeable zinc–copper voltaic battery from simple and cheap electrolyte/separator materials, thus eliminating the need to use the above components. Notably, our battery leverages the Zn_4_SO_4_(OH)_6_·*x*H_2_O precipitation in ZnSO_4_ electrolytes, a common side reaction in zinc batteries, to provide a “locally alkaline” environment for copper electrodes. Consequently, oxide (O^2–^) anion insertion takes place and readily transforms copper to copper(I) oxide (Cu_2_O) without any copper ion dissolution issue. Therefore, this battery realizes a high capacity of ∼370 mA h g^–1^ and a long cycling of ∼500 cycles. Our work provides an innovative approach to stabilize anion insertion in metal electrodes for energy storage.

## 1 Introduction

Batteries play an indispensable role in energy storage and are ubiquitously used in modern society.^1^ Currently, lithium-ion batteries (LIBs) represent state-of-the-art battery technology, which finds wide applications in electronics, electric vehicles, and other areas.^2^ To pursue higher energy density, both academia and industry are actively investigating Li metal and solid-state batteries,^3−5^ which are aimed for long-range electric vehicles. In addition to Li-based batteries, Na, K, and Zn batteries have also gained tremendous attention for stationary energy storage,^6−9^ due to their high abundance and low cost. Overall, the battery field has made substantial progress and will continue to thrive in the following years, due to the increasing demand for renewable energy, carbon neutralization, and emerging applications.^10^

**Figure 1:**
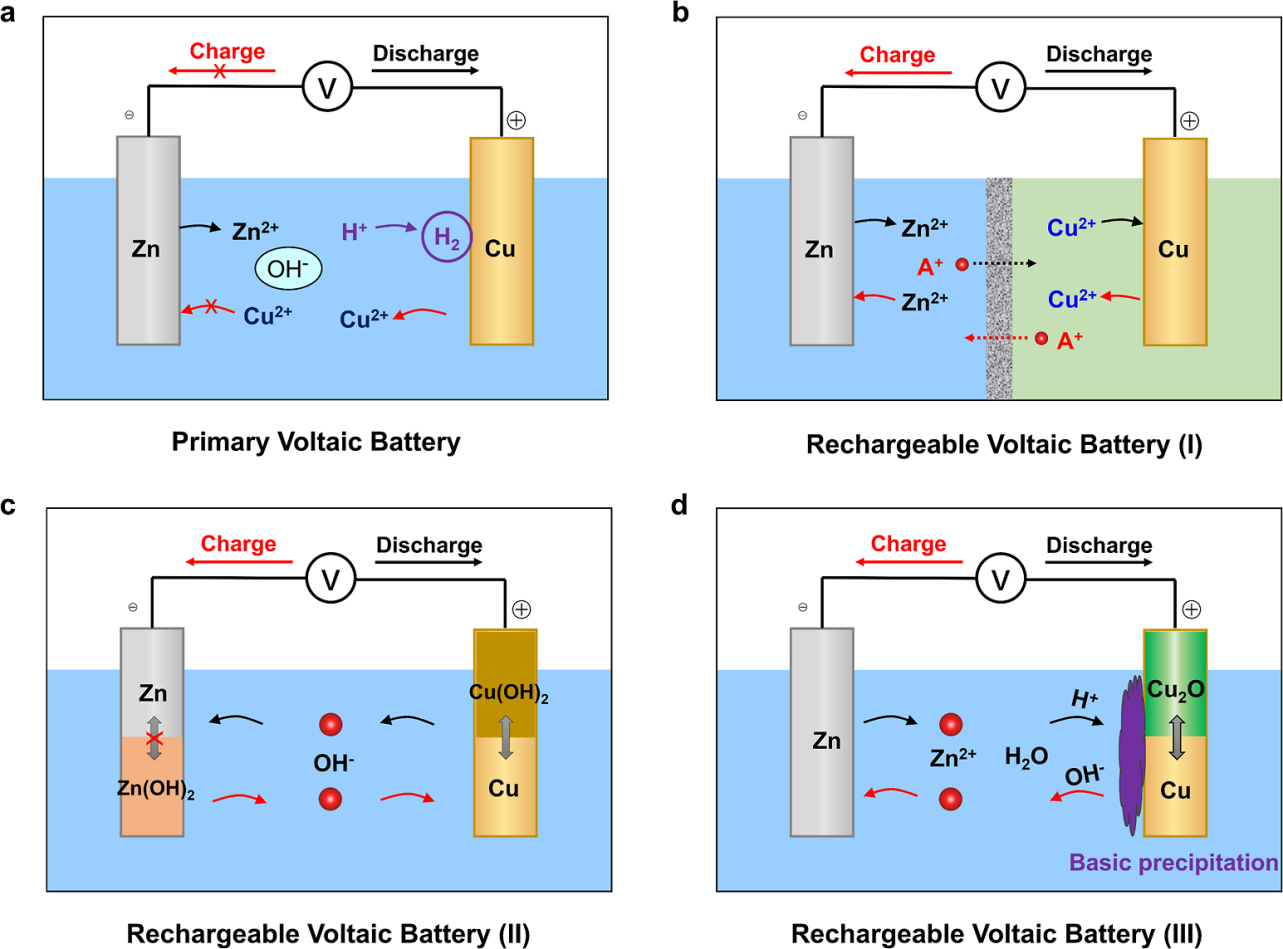
Working mechanisms of voltaic batteries. (a) Primary voltaic battery in NaCl electrolytes; (b) rechargeable voltaic battery that uses a solid-state electrolyte or ion-exchange membrane to physically decouple Zn^2+^/Zn and Cu^2+^/Cu reactions; (c) rechargeable voltaic battery that uses a corrosive alkaline electrolyte to suppress the Cu^2+^ ion solubility; and (d) our proposed rechargeable voltaic battery in a conventional ZnSO_4_ electrolyte, where the alkaline precipitation on copper facilitates the O^2–^ anion insertion.

Big things have small beginnings. Looking back at the battery development history, one would acknowledge the unparalleled invention of the voltaic pile by Alessandro Volta in 1800, which was the very first battery and laid the foundation for the subsequent battery development.^11,12^Figure 1a illustrates its working mechanism,^12^ where two different metals, i.e., zinc and copper, were placed in juxtaposition to serve as the anode and cathode. The electrolyte is a concentrated brine (NaCl) solution, providing some amounts of protons. During the discharge, Zn metal loses electrons and yields Zn^2+^ ions, whereas protons receive electrons and transform into hydrogen gas on the Cu cathode. In this process, Cu does not participate in the redox reaction but acts as a substract for the hydrogen evolution reaction (HER) to take place. If the battery is compelled to charge, then the Cu metal will be oxidized into soluble copper ions, which migrate to the anode side and eventually lead to a spontaneous self-corrosion reaction. Due to these issues, the voltaic pile pertains to a primary battery and cannot charge reversibly.

Despite its crude design and performance, the voltaic battery has a profound impact on the following battery systems, including the Daniell cell (1836), Leclanché cell (1866), zinc–carbon batteries (1886), etc.^12^ Meanwhile, the voltaic battery promotes the development of the electrolysis field,^13−16^ such as decomposition of water and electrochemical plating/separation of metals (Na, K, Ca, etc.). Because of its historic significance, it is of great interest to remedy its working mechanism and revive it as a rechargeable battery. Furthermore, revisiting the voltaic battery is of importance for building high-energy batteries, considering the potentially high capacity of two metal electrodes.

To implement a rechargeable voltaic battery, it is critical to solve the Cu^2+^ ion dissolution and crossover issue. Currently, there are two major approaches in the literature. The first one is to replace the conventional separator with a solid-state electrolyte or an ion-exchange membrane (Figure 1b),^17−20^ which physically decouple Zn^2+^/Zn and Cu^2+^/Cu reactions. To balance the charge, extra cations or anions must be used to commute between the catholyte and anolyte solutions.^17−20^ However, these solid electrolytes and membranes are difficult to manufacture and highly expensive.^21,22^ Moreover, using extra salts and electrolytes dramatically increases the battery mass, which decreases the practical energy density. Additionally, this method makes the battery design complicated to use.

The second approach is to utilize a functional electrolyte, which chemically suppresses the Cu^2+^ ion solubility.^23−25^ For instance, potassium hydroxide electrolytes have been used in Zn–Cu batteries (Figure 1c), which promote the Cu ↔ Cu(OH)_2_ conversion at the cathode.^23^ However, it is well known that KOH, as a strong base, is highly corrosive to the Zn anode.^26−28^ In other words, the KOH strategy stabilizes the Cu cathode performance at the sacrifice of jeopardizing the zinc anode. Most recently, researchers developed a water/ionic liquid biphasic electrolyte to restrict soluble Cu^2+^ ions from diffusing to the zinc anode, leading to excellent cycling life.^25^ Nevertheless, the ionic liquid used in their work is highly toxic and expensive, leading to health and safety concerns.

We aim to build a rechargeable voltaic battery from simple and cheap materials, which avoids using expensive, toxic, or corrosive components. As it is known, there is a pH trade-off between the Cu cathode and Zn anode, where Cu prefers an alkaline condition for conversion reactions,^23,29^ whereas Zn favors a near-neutral solution for plating reactions.^30^ Along this line of thinking, if we can create a “locally alkaline” condition from a near-neutral solution, the pH demands of Cu and Zn should be satisfied simultaneously. We noticed that zinc sulfate hydroxide (Zn_4_SO_4_(OH)_6_) precipitation is a common phenomenon in ZnSO_4_ electrolytes,^30−32^ which contains abundant hydroxide (OH^–^) anions in its structure, thus pertaining to an alkaline substance. Therefore, we may take advantage of this precipitation to mitigate the Cu^2+^ dissolution issue.

Herein, we devise a rechargeable voltaic battery (Figure 1d) using conventional ZnSO_4_ electrolytes and regular separators, which are readily available materials. Particularly, we exploit the in situ Zn_4_SO_4_(OH)_6_ precipitation and coverage on nanosized Cu electrodes to facilitate a solid–solid Cu ↔ Cu_2_O conversion reaction. As a result, the unfavorable Cu^2+^ ion dissolution issue is effectively resolved, and reversible insertion of the O^2–^ takes place, leading to a high capacity of ∼370 mA h g^–1^ and stable cycling of ∼500 cycles.

## 2 Experimental Section

### 2.1 Material Synthesis

The nanosized Cu was synthesized based on a precipitation method. We first made an aqueous solution of CuSO_4_ (0.2 M, 50 mL), and then we added 4 mL of concentrated ammonia solution into the above solution, to make the final NH_3_ concentration 1.2 M. This solution is named solution A. When adding ammonia to the CuSO_4_ solution, light blue precipitation can be observed, which is due to the Cu(OH)_2_ precipitation. After adding all of the ammonia solution, we can get a dark blue solution, which is due to the formation of the [Cu(NH_3_)_4_]^2+^ complex. We also made another solution of NaBH_4_ (50 mL, 0.4 M), solution B, to reduce the copper complex to copper metal. Both A and B solutions need to have pH values of ∼12, which can be adjusted by adding a 1 M NaOH solution. The A solution was purged with N_2_ gas and heated to 40 °C with magnetic stirring, and then we poured the B solution into the A solution in 1 min. We could immediately observe the generation of red precipitates. After 1 h of reaction, these precipitates were washed with water and ethanol many times, which were further dried in a vacuum oven at room temperature. However, even with N_2_ protection, these nanosized copper powders still contain some Cu_2_O impurities. To obtain the high-purity copper powders, we used argon/hydrogen (5% H_2_) to thermally reduce these powders at 300 °C for 20 min. To avoid material oxidization, we stored these Cu powders inside the glovebox.

Copper foil is the one that is used as current collectors for LIBs, which were punched in a circular shape with a 1 cm diameter for use. The micrometer-sized Cu powders were purchased from Sigma-Aldrich. The Zn_0.18_Cu alloy was prepared by mixing Zn and Cu powders in a high-energy ball milling for 4 h, which was well sealed in an argon-filled condition.

### 2.2 Material Characterization

X-ray diffraction (XRD) patterns of the copper materials and copper self-standing electrodes were collected on a Rigaku SuperNova equipped with a HyPix3000 X-ray detector and Cu Kα radiation source (λ = 1.5406 Å). Scanning electron microscopy (SEM) images and energy-dispersive X-ray spectra (EDX) mapping of the copper materials/electrodes were recorded with a field emission scanning electron microscope (JEOL, JSM-6480LV).

### 2.3 Electrode Preparation

The copper foil electrode was directly punched into a circular shape (1 cm in diameter, 9 μm in thickness) for use. The micron-sized and nanosized copper electrodes are sensitive to oxidization, which were made in an argon-filled glovebox (Vigor, O_2_/H_2_O content is lower than 0.5 ppm). Typically, copper powders were ground with Ketjenblack carbon in an 8:1 ratio for 20 min, and then we added PVDF/NMP (PVDF: polyvinylidene fluoride; NMP: *N*-methyl-pyrrolidone) binder solution to make a homogeneous electrode slurry. The slurry was cast on carbon fiber current collectors (Fuel Cell Store, AvCarb MGL370, 0.37 mm thickness and 1 cm in diameter). Then, the electrodes were vacuum-dried inside the antechamber with vacuum pumping running overnight. Therefore, the whole electrode preparation was done inside the glovebox, and the copper electrodes did not make contact with the air, which avoids material oxidization. The mass ratio between the active material, carbon, and binder is 8:1:1, and the active mass loading is 1.5–3.0 mg cm^–2^. For ex situ XRD and SEM tests, the copper powders were ground with Ketjen carbon, and then they were mixed with polytetrafluoroethylene (PTFE) binder emulsion, which were further rolled into a thin self-standing film. The mass ratio is 8:1:1, and the process was done inside the glovebox. To etch the precipitation, we retrieved the nanosized Cu (nano-Cu for short hereafter) electrode from a coin cell at the corresponding state, and we used 1.0 M NH_3_ solution to soak it for 5 min.

Battery assembly and testing: All of these batteries were assembled in a CR2032 coin cell configuration. To mimic the historic voltaic battery, we used copper foil, zinc metal foil, a cloth separator, and saturated NaCl aqueous solution. To make rechargeable voltaic batteries, we used copper electrodes as the cathode, zinc metal foils as the anode, glass fiber separators, and 1.0 M ZnSO_4_ aqueous electrolytes. To prevent the copper from oxidization, the electrolyte was purged by nitrogen gas for 30–60 min and then transferred into the argon-filled glovebox (Vigor, the O_2_/H_2_O content is lower than 0.5 ppm). The battery performance was tested on the Landt Battery System (CT3002AU).

### 2.4 Calculation of the Volume Change between Cu and Cu_2_O

Both Cu and Cu_2_O adopt a cubic structure, and their lattice parameters are 3.615 and 4.2696 Å, respectively. Therefore, the volume of their unit cells can be calculated as 47.24 and 77.83 Å^3^, respectively. The volume change ratio is (77.83 – 47.24)/47.24 = 64.8%.

### 2.5 Calculation of the Electron Transfer Number

The slope capacity is approximately 150 mA h g^–1^. Based on the equation of *C* = 96,485 × *n*/(3.6 × *M*), the electron transfer number (*n*) is calculated as 0.36. In this equation, 96,485, *n*, and *M* represent the Faraday constant, electron transfer number, and molar mass of the electrodes, respectively. Therefore, we will form a Zn_0.18_Cu alloy if Zn^2+^ insertion occurs: 0.18Zn^2+^ 0.36e^–^ + Cu = Zn_0.18_Cu.

## 3 Results and Discussion

**Figure 2:**
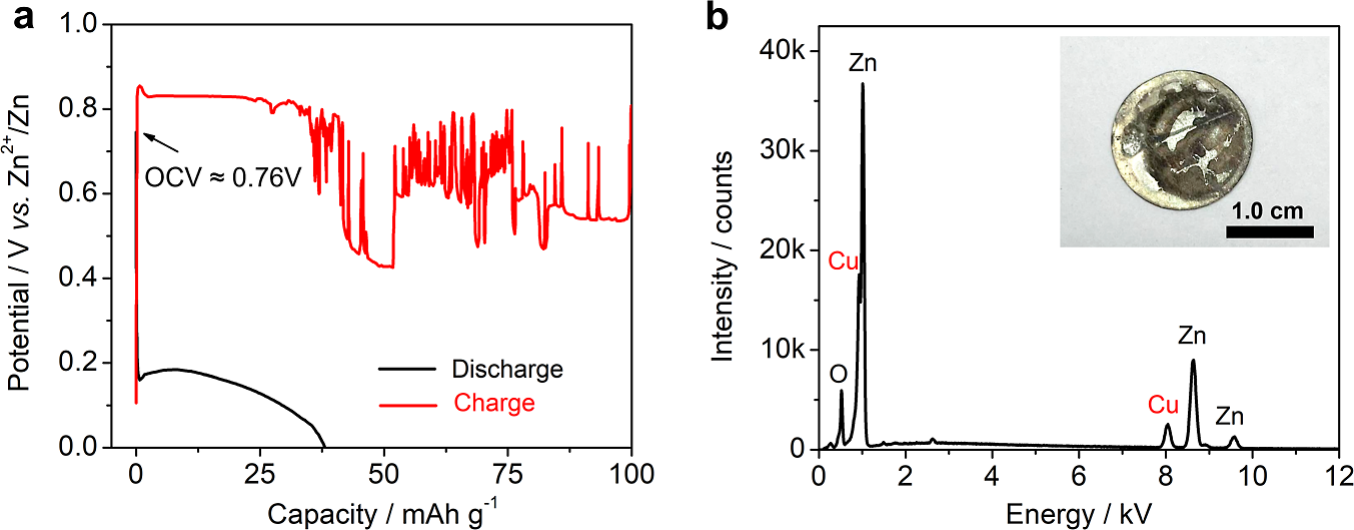
Primary voltaic battery performance. (a) Charge/discharge curve at 20 mA g^–1^ and (b) EDS analysis of the zinc metal; the inset is a digital photograph of zinc (diameter: 1.55 cm).

For a better understanding, we first benchmarked the primary voltaic battery performance, which comprises a Cu foil cathode, a zinc foil anode, and a saturated NaCl electrolyte. As shown in Figure 2a, it exhibits an open-circuit voltage of ∼0.76 V, which results from the low potential of the zinc metal (−0.76 V vs the standard hydrogen electrode). Upon discharge, the cell voltage quickly drops to ∼0.15 V, and the capacity is only ∼37 mA h g^–1^ (Figure 2a), leading to a minimal energy density of 5.6 W h kg^–1^ (based on the Cu mass). Such a low voltage explains the fact that voltaic cells need to be piled up in series for use in history, which should also be the origin of the term “voltaic pile”.

When the battery is subjected to a charging process, the voltage initially increases to ∼0.82 V and then drops to ∼0.5 V with severe fluctuation. After disassembling the cell, we observed colorful deposits on the Zn metal (Figure 2b inset). Energy-dispersive spectroscopy (EDS, Figure 2b) reveals the presence of the Cu element on the Zn surface, which confirms the Cu^2+^ ion dissolution and crossover issue. These results prove that the primary voltaic battery exhibits poor performance, with a low operation voltage, a low capacity, and nonrechargeability.

**Figure 3:**
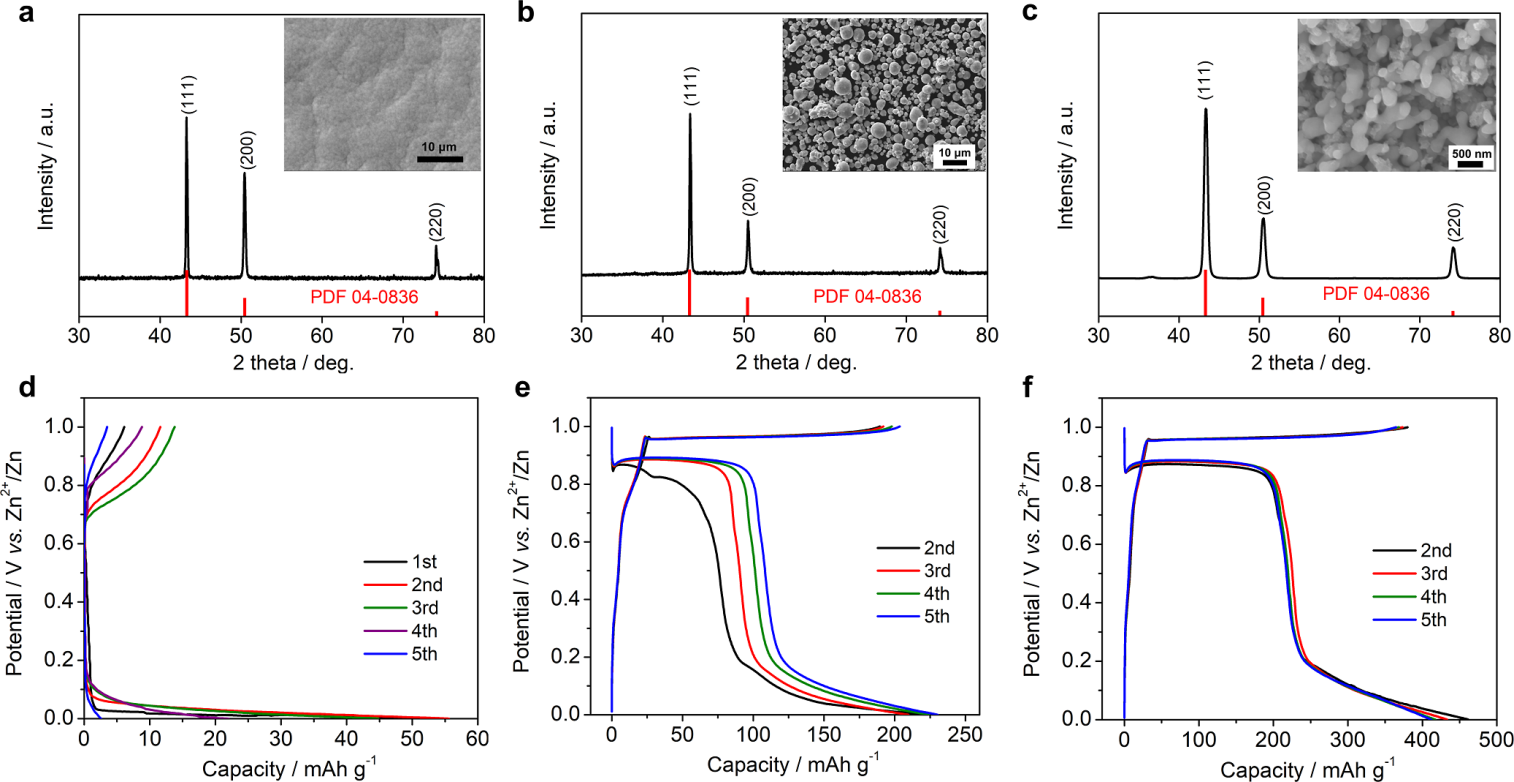
Physical and electrochemical characterization of Cu metals. The XRD patterns of the bulk Cu foil (a), micrometer-sized Cu (b), and nanosized Cu (c). Their corresponding SEM images are given in the inset. The GCD curves of the bulk Cu foil (d), micron-sized Cu (e), and nanosized Cu (f) in 1 M ZnSO_4_ electrolytes. The current density is 20 mA g^–1^.

We utilized the conventional 1 M ZnSO_4_ electrolyte and regular glass fiber separators to assemble the 2032-type coin cells. Three Cu metals were used for a systematic comparison, i.e., bulk foil, micron-sized powder, and nanosized powder. The bulk Cu foil is the one used as the LIB current collectors. The micrometer-sized Cu was purchased from Sigma-Aldrich. The nanosized Cu was prepared by a chemical method.^33^Figure 3a–c shows their XRD patterns and SEM images. All of these samples exhibit high purity, and XRD patterns are well indexed to a cubic Cu phase (PDF: 04-0836, space group: *Fm*3̅*m*, *a* = 3.615 Å, α = 90°). The Cu foil has a relatively smooth surface, and the micrometer-sized Cu exhibits spherical morphology with a size distribution of 0.5–5 μm. The nanosized Cu powders appear to be quasi-spherical, and the particle size ranges from 50 to 300 nm.

Figure 3d–f shows the galvanostatic charge/discharge (GCD) curves of these Zn–Cu batteries in 1 M ZnSO_4_. The Cu foil delivers an initial discharge/charge capacity of ∼55/11 mA h g^–1^, and the capacity fades to <5 mA h g^–1^ in the following cycles (Figure 3d). Despite the low capacity and poor cycling, the Cu foil manages to support several GCD cycles, which surpasses that of the primary voltaic battery in NaCl electrolytes (Figure 2a). Therefore, replacing the electrolyte is beneficial to increase the reaction reversibility. Figure 3e shows the micrometer-sized Cu performance, which exhibits well-defined GCD curves, with one plateau during charge, and a plateau plus a slope during discharge. The reversible capacity stabilizes at ∼200 mA h g^–1^.

In comparison, the nano-Cu exhibits similar GCD curves to micron-Cu, but it delivers an even higher charge capacity of ∼370 mA h g^–1^ (Figure 3f). It is likely that its smaller particle size and faster reaction kinetics lead to improved capacity utilization. The GCD curves overlap well, indicating a reversible redox reaction. Of note, the charge capacity (∼370 mA h g^–1^) is an order of magnitude higher than that in primary voltaic batteries (37 mA h g^–1^, Figure 2b). This capacity is also on par with common Zn-ion cathode materials, such as metal oxides^34−38^ and organic materials.^39^ Besides, the middle-capacity voltage is ∼0.85 V, exceeding the primary battery (0.15 V) by 0.7 V. This voltage is also reasonable in the aqueous battery context, sufficient for direct use without piling up. It is noted that the initial GCD curves of micron-Cu and nano-Cu electrodes are provided in Figure S1, where ultrahigh discharge capacities (1000–1200 mA h g^–1^) are observed. As will be discussed, this capacity results from catalytic hydrogen evolution and Zn_4_SO_4_(OH)_6_·H_2_O formation. We need to note that the potential gap between the charge and discharge is relatively large (Figure 3e,f), which leads to a substantial polarization and suboptimal round-trip energy efficiency (∼60%).

**Figure 4:**
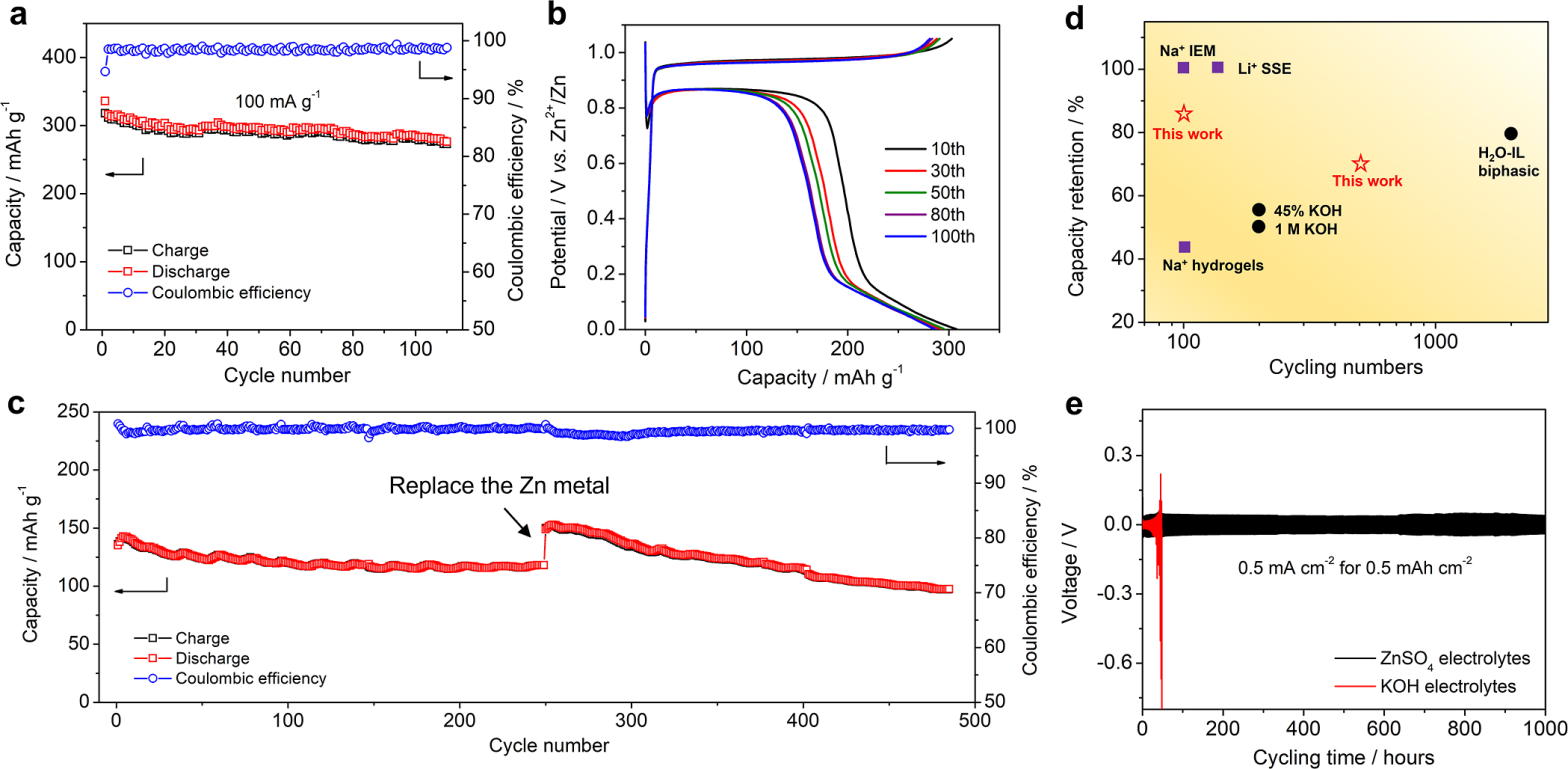
Cycling performance of our voltaic battery. (a) Cycling performance at 100 mA g^–1^; (b) selected GCD curves during cycling at 100 mA g^–1^; (c) cycling performance at 200 mA g^–1^; (d) cycling comparison of our battery with other rechargeable voltaic batteries; and (e) GCD curves of symmetrical Zn∥Zn batteries in ZnSO_4_ and KOH electrolytes.

Besides the high capacity, our voltaic battery also demonstrates an excellent cycling performance. At 100 mA g^–1^, the charge capacity fades from 318 to 272 mA h g^–1^ (Figure 4a), corresponding to a good capacity retention of 85% and an average Coulombic efficiency of 98.5%. Figure 4b displays the selected GCD curves, which are well overlapped, suggesting a highly reversible insertion reaction. At 200 mA g^–1^ current density (Figure 4c), the overall capacity retention is 71% after 485 cycles and the average Coulombic efficiency is 99.6%. Note that there was a cell short circuit due to the power surge at the 250th cycle, and we retrieved the copper electrode to make a new battery with a new Zn metal. The selected GCD curves during cycling are shown in Figure S2. The battery rate capability is shown in Figure S3.

Compared with the literature results, our battery showcases an outstanding cycling performance (Figure 4d), although we did not use any sophisticated cell components or electrolytes. When Li-based solid-state electrolytes (SSEs) and Na-based ion-exchange membrane (IEM) were used,^17,18^ the voltaic battery showed high-capacity retention of ∼100%; however, their cycling numbers are limited to 100–150 cycles. Meanwhile, the SSE or IEM is hard to manufacture and is highly expensive, which markedly limits the practical applications. When Na^+^ hydrogel electrolytes were used as cheaper alternatives,^19^ the cycling performance was largely compromised, with only 43% capacity retention after 100 cycles. In our case, the battery can cycle for 485 cycles with 71% retention.

On the other hand, using functional electrolytes can prolong the cycling numbers, but these electrolytes incur other issues. For instance, the KOH electrolyte renders a longer cycling number to 200 cycles,^23,24^ but the capacity retention is less than 50%. It is likely that Zn metal anode corrosion exacerbates the cycling stability. As shown in Figure 4e, the ZnSO_4_ electrolyte renders stable cycling in Zn∥Zn symmetrical batteries for 1000 h, but the KOH electrolyte leads to a severe polarization increase after merely 35 h. Most recently, a water/ionic liquid biphasic electrolyte was developed for voltaic batteries,^25^ which shows a remarkable capacity retention of 80% after 2000 cycles. However, the ionic liquid of 1-ethyl-3-methylimidazolium bis(trifluoromethylsulfonyl)imide used in their work is highly expensive and has acute toxicity. Besides, they still need to use an expensive ion-exchange membrane to make full cells. Table S1 provides a more detailed comparison of these works.

**Figure 5:**
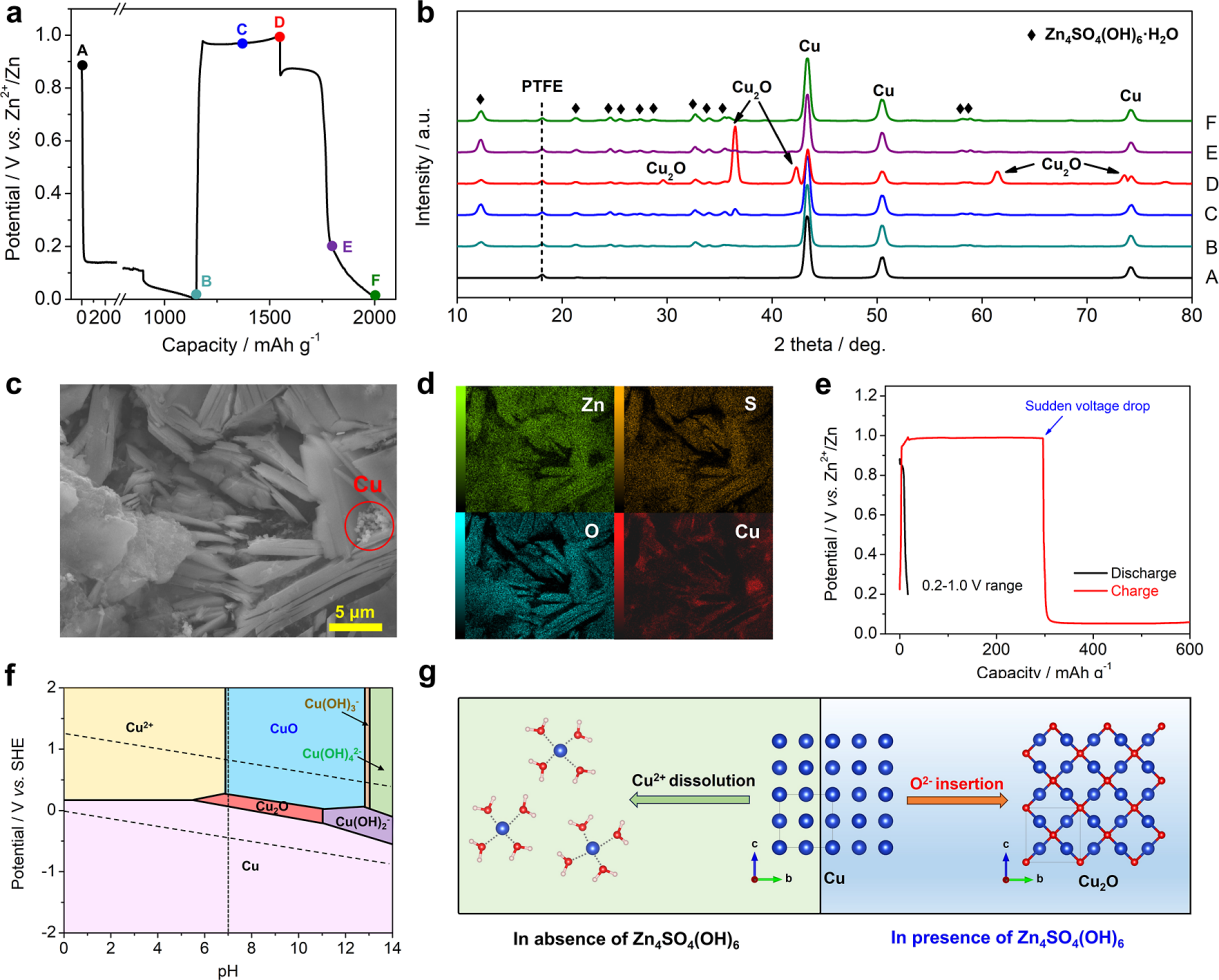
Mechanism studies of the rechargeable voltaic batteries. (a) Selected charge/discharge states; (b) ex situ XRD patterns at corresponding GCD states; (c,d) SEM images and elemental mapping results of point B; (e) GCD curves of the nano-Cu electrode in a narrower potential range of 0.2–1.0 V; and (f) pH-potential diagram of the Cu–H_2_O system at 298 K, where the Cu^2+^ concentration is 10^–6^ mol kg^–1^. Reproduced with permission from ref (40). Copyright 1997, IOP Publishing. (g) Scheme of the Cu^2+^ ion dissolution and the Cu → Cu_2_O solid–solid conversion.

To understand the Cu cathode reaction mechanism, we carried out ex situ XRD tests on nano-Cu electrodes at different GCD states, where six representative states were selected (Figure 5a), where points A–F represent the pristine, initial full discharge, half charge, full charge, discharge to 0.2 V, and full discharge, respectively.

During the initial discharge (point B), we observed a high discharge capacity of ∼1150 mA h g^–1^; however, there is no peak shift or intensity change for the nano-Cu electrode. Instead, there are multiple peaks at 12.3, 21.4, 24.6, 27.6, 32.7, and 34.1° (Figures 5b and S4), which are well attributed to the Zn_4_SO_4_(OH)_6_·H_2_O phase. Therefore, there is a significant HER in the initial discharge, which consumes H^+^ ions and generates OH^–^ ions, leading to Zn_4_SO_4_(OH)_6_·H_2_O precipitation. SEM and EDS elemental mapping further corroborate the XRD result. As shown in Figure 5c,d, layer-structured and micrometer-sized flakes are evident in the electrode, with Zn, S, and O elements well distributed, thus confirming the Zn_4_SO_4_(OH)_6_·H_2_O formation. Besides, nano-Cu powders are surrounded and well encapsulated by the Zn_4_SO_4_(OH)_6_·H_2_O precipitation (Figures 5d and S5), which will be beneficial for the following Cu ↔ Cu_2_O conversion. The initial discharge reactions are proposed as
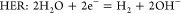
1
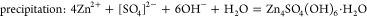
2
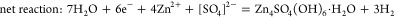
3

During the charging process, the nano-Cu electrode progressively transforms to Cu_2_O, with characteristic peaks emerging at 29.5, 36.3, 42.2, 61.4, and 73.5° (Cu_2_O, PDF 05-0667, cubic, *Pn*3̅*m*, *a* = *b* = *c* = 4.2696 Å, α = 90°), as shown in Figures 5b and S4. At the fully charged state (point D), the electrode is a mixture of Cu and Cu_2_O, indicating a less complete conversion reaction. If Cu is fully converted to Cu_2_O, then a high charge capacity of ∼421 mA h g^–1^ will be achieved.

We underline that the Zn_4_SO_4_(OH)_6_·H_2_O phase is present during the entire conversion process, which is critical for Cu ↔ Cu_2_O conversion. If we subject the nano-Cu electrode to a narrower potential range (0.2–1.0 V) and prevent the Zn_4_SO_4_(OH)_6_·H_2_O formation, the nano-Cu electrode will suffer from a sudden potential drop when the capacity reaches ∼300 mA h g^–1^ (Figure 5e). We thus disassembled the cell and observed red deposits on the Zn metal surface (Figure S6). EDS analysis reveals the presence of the Cu element on the Zn anode, which validates the Cu^2+^ ion dissolution and crossover process (Figure S6). Therefore, it casts a critical question: Why does pure Cu suffer from Cu^2+^ dissolution (Figure 5e), but the Cu electrode embedded in Zn_4_SO_4_(OH)_6_·H_2_O successfully transforms to the insoluble Cu_2_O phase without any issue?

We believe that their performance discrepancy can be explained by the pH-potential diagram of the Cu–H_2_O system (Figure 5f).^40^ The ZnSO_4_ electrolyte has a near-neutral pH value (pH ≈ 5), which favors the Cu (s) → Cu^2+^ (aq) reaction upon oxidization. This accounts for Cu^2+^ dissolution in the pure Cu case (Figure 5g). By contrast, the Zn_4_SO_4_(OH)_6_·H_2_O material contains abundant hydroxide in its structure, which can be released due to the solubility equilibrium effect. Therefore, it can act as an OH^–^ reservoir to maintain a locally alkaline pH condition for Cu, thus facilitating Cu → Cu_2_O conversion (Figure 5g). Figure S7 shows that the Cu/Cu_2_O electrode is also well dispersed in the Zn_4_SO_4_(OH)_6_·H_2_O matrix in the fully charged state. Therefore, the reactions during charging are proposed as follows
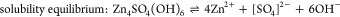
4
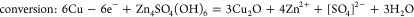
5

Notably, both Cu and Cu_2_O adopt a cubic phase (Figure 5g), with high structural similarity. After the O^2–^ anion insertion, the lattice parameter *a* increases from 3.615 to 4.2696 Å, which corresponds to a moderate volume increase ratio of 65% (see the calculation in the Experimental Section). This ratio is even smaller than some conversion cathodes in LIBs, such as Co_3_O_4_ (99.0%), CoO (84.2%), and Fe_3_O_4_ (80.3%).^41^ Hence, the Cu_2_O/Cu structural similarity and moderate volume change facilitate battery cycling.

During the subsequent discharge process (points D–F), the Cu_2_O peaks entirely disappear, and the Cu peak intensity gets intensified, confirming the Cu_2_O → Cu reduction process (Figure 5b). The reaction is expressed as
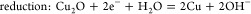
6

**Figure 6:**
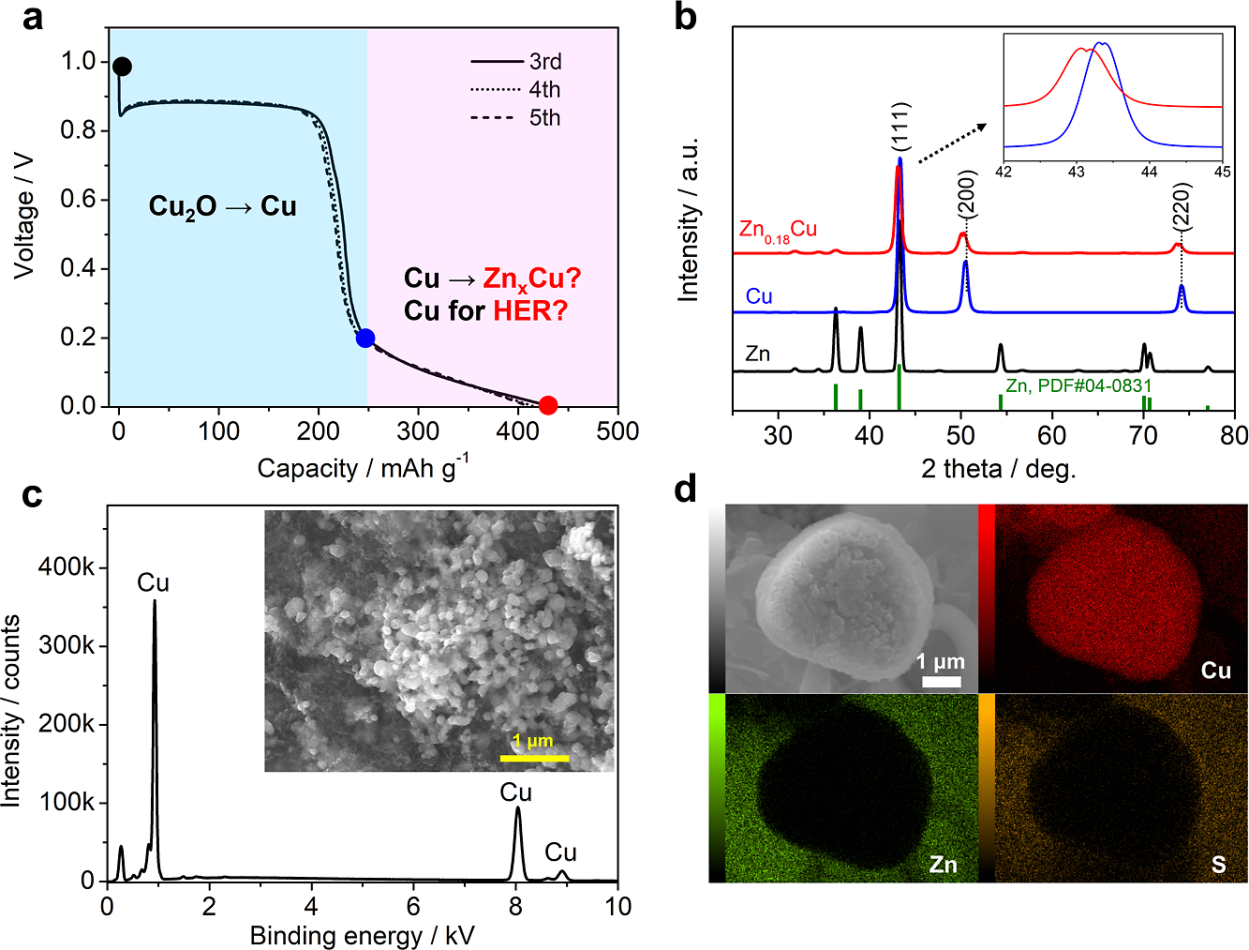
(a) GCD curves of the nano-Cu electrodes at 20 mA g^–1^; (b) XRD patterns of the pure Cu, pure Zn, and synthesized Zn_0.18_Cu alloy; (c) EDS result of the ammonia-etched nano-Cu electrode (fully discharged), the inset is a SEM image; (d) EDS mapping result of the micron-sized Cu electrode at the fully discharged state.

The Cu ↔ Cu_2_O solid–solid conversion explains the reaction plateau well; however, the discharge slope in the 0–0.2 V range remains elusive. Particularly, this slope appears to be very repeatable and shows up in each cycle (Figures 6a and 4b), which provides an appreciable capacity of ∼150 mA h g^–1^. Furthermore, this process is also quite reversible because the Coulombic efficiency is 98.5% (Figure 4b), close to 100%. However, based on the ex situ XRD result (point E to F, Figure 5b), there is no generation of new peaks or a peak shift in the Cu electrode. Hence, a crucial question arises: What redox reaction contributes to this reversible slope? In the ZnSO_4_ electrolyte, there are only two cations (Zn^2+^ and H^+^), therefore, only two possible reactions should occur: (1) reversible Zn^2+^ insertion in Cu to form a Zn_*x*_Cu alloy and (2) reversible H^+^ reduction to a hydrogen gas (Figure 6a). The second reaction seems unlikely and surprising at first glance, but it should be the most reasonable explanation in this work.

Based on the slope capacity of ∼150 mA h g^–1^, the electron transfer number is calculated as 0.36 mol per copper (see the calculation in the Experimental Section). Thus, if we assume Zn^2+^ insertion, we will form a Zn_0.18_Cu alloy in the fully discharged state. We experimentally prepared a Zn_0.18_Cu alloy material by mixing Zn and Cu powders via high-energy ball milling. However, this Zn_0.18_Cu alloy exhibits a structure expansion compared with nano-Cu powders, because the (111), (200), and (220) planes shift to a lower diffraction angle (Figure 6b). In the ex situ XRD pattern (Figures 5b and S8), there is no peak shift at all for the nano-Cu electrode. Thus, the XRD results do not support the alloy formation.

SEM and EDS characterizations also rule out Zn_0.18_Cu alloy formation. If Zn^2+^ insertion takes place, then the Cu electrode will contain a noticeable amount of Zn at the fully discharged state. Unfortunately, the Zn_4_SO_4_(OH)_6_ precipitation interferes with the verification. Therefore, we retrieved the fully discharged nano-Cu electrode and used an ammonia solution to wash away the Zn_4_SO_4_(OH)_6_ precipitation. As shown in Figure 6c, the etched Cu electrode does not have any micrometer-sized flakes, and EDS analysis detects the Cu element only. This indicates that there is no alloy formation during the discharge. Of note, Zn^2+^ ions in the Zn_4_SO_4_(OH)_6_ precipitation will be etched by NH_3_, due to the [Zn(NH_3_)_4_]^2+^ complex ion formation; however, the Zn element in the hypothetical alloy (Zn_*x*_Cu) will not be etched by the NH_3_ solution, because NH_3_ is not an oxidizing agent.

To further corroborate the nano-Cu result, we also characterized the micrometer-sized Cu electrode, which shows a similar GCD curve to nano-Cu but has a much larger particle size for straightforward observation. Figure 6d shows the SEM image and the EDS mapping of the micrometer-sized Cu at the fully discharged state. Evidently, the Zn element is separated from the Cu element, but it overlaps with the S element well. This means that all of the Zn signals come from the Zn_4_SO_4_(OH)_6_ precipitation other than the hypothetical Zn_*x*_Cu alloy.

Based on the above results, we propose that the 0–0.2 V slope results from a quasi-reversible HER, which is triggered by the nano-Cu, leading to a high discharge capacity (∼150 mA h g^–1^). Besides, the H_2_ gas could be trapped, confined, or absorbed by numerous Zn_4_SO_4_(OH)_6_ flakes, thus participating in the charging process. This explains that the Coulombic efficiency (charge capacity divided by discharge) is quite high during cycling (∼98.5%, Figure 4b), and there is a minimal change in ex situ XRD patterns (point B to C, point E to F, Figure 5b). We understand that this statement may sound incredible, but as it goes, “once we have eliminated the impossible, whatever remains, however improbable, must be the truth”. More advanced and sophisticated characterizations will be carried out to further understand this slope reaction.

## 4 Conclusions

In summary, we demonstrate a rechargeable voltaic battery using a simple and cost-effective ZnSO_4_ electrolyte. Due to the precipitation of zinc sulfate hydroxide, the copper electrode is well dispersed in this alkaline substance matrix, which effectively avoids Cu^2+^ ion dissolution. Consequently, the insertion of the O^2–^ anion readily takes place, thus transforming Cu to Cu_2_O. Therefore, the battery achieves a high capacity of ∼370 mA h g^–1^ and stable cycling of ∼500 cycles. Our battery design efficiently balances the pH needs between the Cu cathode and the Zn anode, and it eliminates the need to use expensive and sophisticated cell components, including solid electrolytes, ion-exchange membranes, and corrosive/toxic electrolytes. This work offers insights on how to stabilize anion insertion for energy storage.

## Supporting Information Available

Experimental details on the material/electrode preparation, and physical and electrochemical characterization (XRD, TGA, SEM, and charge/discharge curves) (PDF)

## References

[ref1] Hu Y.-S.; Lu Y. 2019 Nobel Prize for the Li-Ion Batteries and New Opportunities and Challenges in Na-Ion Batteries. ACS Energy Lett. 2019, 4 (11), 2689–2690. 10.1021/acsenergylett.9b02190.

[ref2] Goodenough J. B.; Park K.-S. The Li-Ion Rechargeable Battery: A Perspective. J. Am. Chem. Soc. 2013, 135 (4), 1167–1176. 10.1021/ja3091438. 23294028

[ref3] Cheng L.; Ma C.; Lu W.; Wang X.; Yue H.; Zhang D.; Xing Z. A Graphitized Hierarchical Porous Carbon as an Advanced Cathode Host for Alkali Metal-Selenium Batteries. Chem. Eng. J. 2022, 433, 133527 10.1016/j.cej.2021.133527.

[ref4] Lin B.; Zhang Y.; Li W.; Huang J.; Yang Y.; Or S. W.; Xing Z.; Guo S. Recent Advances in Rare Earth Compounds for Lithium-Sulfur Batteries. eScience 2023, 100180 10.1016/j.esci.2023.100180.

[ref5] Gao N.; Li B.; Zhang Y.; Li W.; Li X.; Zhao J.; Yue W.; Xing Z.; Wang B. CoFe Alloy-Decorated Interlayer with a Synergistic Catalytic Effect Improves the Electrochemical Kinetics of Polysulfide Conversion. ACS Appl. Mater. Interfaces 2021, 13 (48), 57193–57203. 10.1021/acsami.1c17374. 34797970

[ref6] Liu Y.; Wu X.; Moeez A.; Peng Z.; Xia Y.; Zhao D.; Liu J.; Li W. Na-Rich Na_3_V_2_ (PO_4_)_3_ Cathodes for Long Cycling Rechargeable Sodium Full Cells. Adv. Energy Mater. 2023, 13 (3), 2203283 10.1002/aenm.202203283.

[ref7] Wu X.; Qiu S.; Liu Y.; Xu Y.; Jian Z.; Yang J.; Ji X.; Liu J. The Quest for Stable Potassium-Ion Battery Chemistry. Adv. Mater. 2022, 34 (5), 2106876 10.1002/adma.202106876. 34648671

[ref8] Chang H. J.; Rodríguez-Pérez I. A.; Fayette M.; Canfield N. L.; Pan H.; Choi D.; Li X.; Reed D. Effects of Water-Based Binders on Electrochemical Performance of Manganese Dioxide Cathode in Mild Aqueous Zinc Batteries. Carbon Energy 2021, 3 (3), 473–481. 10.1002/cey2.84.

[ref9] Dai Y.; Li J.; Zhang C.; Lu R.; Tao X.; Owusu K. A.; He G.; Zhou Y.; Lu J. Fluorinated Interphase Enables Reversible Zn^2+^ Storage in Aqueous ZnSO_4_ Electrolytes. ACS Energy Lett. 2023, 8 (11), 4762–4767. 10.1021/acsenergylett.3c01737.

[ref10] Liu J.; Huang Z.; Fan M.; Yang J.; Xiao J.; Wang Y. Future Energy Infrastructure, Energy Platform and Energy Storage. Nano Energy 2022, 104, 107915 10.1016/j.nanoen.2022.107915.

[ref11] Sudduth W. M. The Voltaic Pile and Electro-Chemical Theory in 1800. Ambix 1980, 27 (1), 26–35. 10.1179/amb.1980.27.1.26.

[ref12] Sarma D. D.; Shukla A. K. Building Better Batteries: A Travel Back in Time. ACS Energy Lett. 2018, 3, 2841–2845. 10.1021/acsenergylett.8b01966.

[ref13] Russell C. A. The Electrochemical Theory of Sir Humphry Davy: Part I: The Voltaic Pile and Electrolysis. Ann. Sci. 1959, 15, 1–13. 10.1080/00033795900200018.

[ref14] Fabbrizzi L. Strange Case of Signor Volta and Mister Nicholson: How Electrochemistry Developed as a Consequence of an Editorial Misconduct. Angew. Chem., Int. Ed. 2019, 58, 5810–5822. 10.1002/anie.201813519. 30773768

[ref15] Decker F. Volta and the Pile. Electrochemistry Encyclopedia; The Electrochemical Society, 2005.

[ref16] Clarke T. B.; Glasscott M. W.; Dick J. E. The Role of Oxygen in the Voltaic Pile. J. Chem. Educ. 2021, 98, 2927–2936. 10.1021/acs.jchemed.1c00016.

[ref17] Dong X.; Wang Y.; Xia Y. Re-Building Daniell Cell with a Li-Ion Exchange Film. Sci. Rep. 2014, 4 (1), 6916 10.1038/srep06916. 25369833 PMC4220274

[ref18] Jameson A.; Khazaeli A.; Barz D. P. J. A Rechargeable Zinc Copper Battery Using a Selective Cation Exchange Membrane. J. Power Sources 2020, 453, 227873 10.1016/j.jpowsour.2020.227873.

[ref19] Mypati S.; Khazaeli A.; Barz D. P. J. A Novel Rechargeable Zinc-Copper Battery without a Separator. J. Energy Storage 2021, 42, 103109 10.1016/j.est.2021.103109.

[ref20] He Z.; Guo J.; Xiong F.; Tan S.; Yang Y.; Cao R.; Thompson G.; An Q.; De Volder M.; Mai L. Re-Imagining the Daniell Cell: Ampere-Hour-Level Rechargeable Zn-Cu Batteries. Energy Environ. Sci. 2023, 16, 5832–5841. 10.1039/D3EE02786D. 38076637 PMC10698845

[ref21] Jiang S.; Sun H.; Wang H.; Ladewig B. P.; Yao Z. A Comprehensive Review on the Synthesis and Applications of Ion Exchange Membranes. Chemosphere 2021, 282, 130817 10.1016/j.chemosphere.2021.130817. 34091294

[ref22] Schnell J.; Tietz F.; Singer C.; Hofer A.; Billot N.; Reinhart G. Prospects of Production Technologies and Manufacturing Costs of Oxide-Based All-Solid-State Lithium Batteries. Energy Environ. Sci. 2019, 12 (6), 1818–1833. 10.1039/C8EE02692K.

[ref23] Zhu Q.; Cheng M.; Zhang B.; Jin K.; Chen S.; Ren Z.; Yu Y. Realizing a Rechargeable High-Performance Cu-Zn Battery by Adjusting the Solubility of Cu^2+^. Adv. Funct. Mater. 2019, 29 (50), 1905979 10.1002/adfm.201905979.

[ref24] Arnot D. J.; Schorr N. B.; Kolesnichenko I. V.; Lambert T. N. Rechargeable Alkaline Zn-Cu Batteries Enabled by Carbon Coated Cu/Bi Particles. J. Power Sources 2022, 529, 231168 10.1016/j.jpowsour.2022.231168.

[ref25] Xu C.; Lei C.; Li J.; He X.; Jiang P.; Wang H.; Liu T.; Liang X. Unravelling Rechargeable Zinc-Copper Batteries by a Chloride Shuttle in a Biphasic Electrolyte. Nat. Commun. 2023, 14 (1), 2349 10.1038/s41467-023-37642-2. 37095106 PMC10125991

[ref26] Dirkse T. P.; Timmer R. The Corrosion of Zinc in KOH Solutions. J. Electrochem. Soc. 1969, 116 (2), 162 10.1149/1.2411786.

[ref27] Suresh Kannan A.; Muralidharan S.; Sarangapani K. B.; Balaramachandran V.; Kapali V. Corrosion and Anodic Behaviour of Zinc and Its Ternary Alloys in Alkaline Battery Electrolytes. J. Power Sources 1995, 57 (1–2), 93–98. 10.1016/0378-7753(95)02225-2.

[ref28] Cai Z.; Wang J.; Sun Y. Anode Corrosion in Aqueous Zn Metal Batteries. eScience 2023, 3, 100093 10.1016/j.esci.2023.100093.

[ref29] Gallagher T. C.; Wu C. Y.; Lucero M.; Sandstrom S. K.; Hagglund L.; Jiang H.; Stickle W.; Feng Z.; Ji X. From Copper to Basic Copper Carbonate: A Reversible Conversion Cathode in Aqueous Anion Batteries. Angew. Chem., Int. Ed. 2022, 61 (31), e202203837 10.1002/anie.202203837. 35522947

[ref30] Pan H.; Shao Y.; Yan P.; Cheng Y.; Han K. S.; Nie Z.; Wang C.; Yang J.; Li X.; Bhattacharya P.; et al. Reversible Aqueous Zinc/Manganese Oxide Energy Storage from Conversion Reactions. Nat. Energy 2016, 1 (5), 16039 10.1038/nenergy.2016.39.

[ref31] Chen H.; Dai C.; Xiao F.; Yang Q.; Cai S.; Xu M.; Fan H. J.; Bao S. J. Reunderstanding the Reaction Mechanism of Aqueous Zn-Mn Batteries with Sulfate Electrolytes: Role of the Zinc Sulfate Hydroxide. Adv. Mater. 2022, 34 (15), 2109092 10.1002/adma.202109092. 35137465

[ref32] Lim W. G.; Li X.; Reed D. Understanding the Role of Zinc Hydroxide Sulfate and Its Analogues in Mildly Acidic Aqueous Zinc Batteries: A Review. Small Methods 2023, 2300965 10.1002/smtd.202300965. 37803913

[ref33] Liu Q.-m.; Zhou D.-b.; Yamamoto Y.; Ichino R.; Okido M. Preparation of Cu Nanoparticles with NaBH_4_ by Aqueous Reduction Method. Trans. Nonferrous Met. Soc. China 2012, 22 (1), 117–123. 10.1016/S1003-6326(11)61149-7.

[ref34] Sun W.; Wang F.; Hou S.; Yang C.; Fan X.; Ma Z.; Gao T.; Han F.; Hu R.; Zhu M.; et al. Zn/MnO_2_ Battery Chemistry with H^+^ and Zn^2+^ Coinsertion. J. Am. Chem. Soc. 2017, 139 (29), 9775–9778. 10.1021/jacs.7b04471. 28704997

[ref35] Li Y.; Huang Z.; Kalambate P. K.; Zhong Y.; Huang Z.; Xie M.; Shen Y.; Huang Y. V_2_O_5_ Nanopaper as a Cathode Material with High Capacity and Long Cycle Life for Rechargeable Aqueous Zinc-Ion Battery. Nano Energy 2019, 60, 752–759. 10.1016/j.nanoen.2019.04.009.

[ref36] Wu J.; Meng J.; Yang Z.; Chen H.; Rong Y.; Deng L.; Fu Z. Energy Storage Mechanism and Electrochemical Performance of Cu_2_O/rGO as Advanced Cathode for Aqueous Zinc Ion Batteries. J. Alloys Compd. 2022, 895, 162653 10.1016/j.jallcom.2021.162653.

[ref37] Schorr N. B.; Arnot D. J.; Bruck A. M.; Duay J.; Kelly M.; Habing R. L.; Ricketts L. S.; Vigil J. A.; Gallaway J. W.; Lambert T. N. Rechargeable Alkaline Zinc/Copper Oxide Batteries. ACS Appl. Energy Mater. 2021, 4 (7), 7073–7082. 10.1021/acsaem.1c01133.

[ref38] Hao J.; Yuan L.; Johannessen B.; Zhu Y.; Jiao Y.; Ye C.; Xie F.; Qiao S. Z. Studying the Conversion Mechanism to Broaden Cathode Options in Aqueous Zinc-Ion Batteries. Angew. Chem. 2021, 133 (47), 25318–25325. 10.1002/ange.202111398. 34553459

[ref39] Shi H. Y.; Ye Y. J.; Liu K.; Song Y.; Sun X. A Long-Cycle-Life Self-Doped Polyaniline Cathode for Rechargeable Aqueous Zinc Batteries. Angew. Chem. 2018, 130 (50), 16597–16601. 10.1002/ange.201808886. 30307094

[ref40] Beverskog B.; Puigdomenech I. Revised Pourbaix Diagrams for Copper at 25 to 300 °C. J. Electrochem. Soc. 1997, 144 (10), 3476–3483. 10.1149/1.1838036.

[ref41] Ko Y.-D.; Kang J.-G.; Choi K. J.; Park J.-G.; Ahn J.-P.; Chung K. Y.; Nam K.-W.; Yoon W.-S.; Kim D.-W. High Rate Capabilities Induced by Multi-Phasic Nanodomains in Iron-Substituted Calcium Cobaltite Electrodes. J. Mater. Chem. 2009, 19 (13), 1829–1835. 10.1039/b817120c.

