# Supplementary material for: Building a Rechargeable Voltaic Battery via Reversible Oxide Anion Insertion in Copper Electrodes
Source: ACS Appl Energy Mater. 2024 Feb 23;7(5):2048–56. doi: 10.1021/acsaem.4c00008 (PMC11033868; doi:10.1021/acsaem.4c00008)
Supplement: Supplementary file 1 — ae4c00008_si_001.pdf [file ae4c00008_si_001.pdf]

Supporting Information for

Building a Rechargeable Voltaic Battery *Via* Reversible Oxide Anion Insertion in Copper Electrodes

Jose Fernando Florez Gomez,[1] Nischal Oli,[1] Songyang Chang,[2] Shen Qiu,[2] Swati Katiyar,[2] Ram Katiyar,[1] Gerardo Morell,[1] and Xianyong Wu*[2]*

[1]Department of Physics, University of Puerto Rico-Rio Piedras Campus, San Juan, Puerto Rico 00925-2537, United States

E-mail: gerardo.morell@upr.edu

[2]Department of Chemistry, University of Puerto Rico-Rio Piedras Campus, San Juan, Puerto Rico 00925-2537, United States

E-mail: xianyong.wu@upr.edu

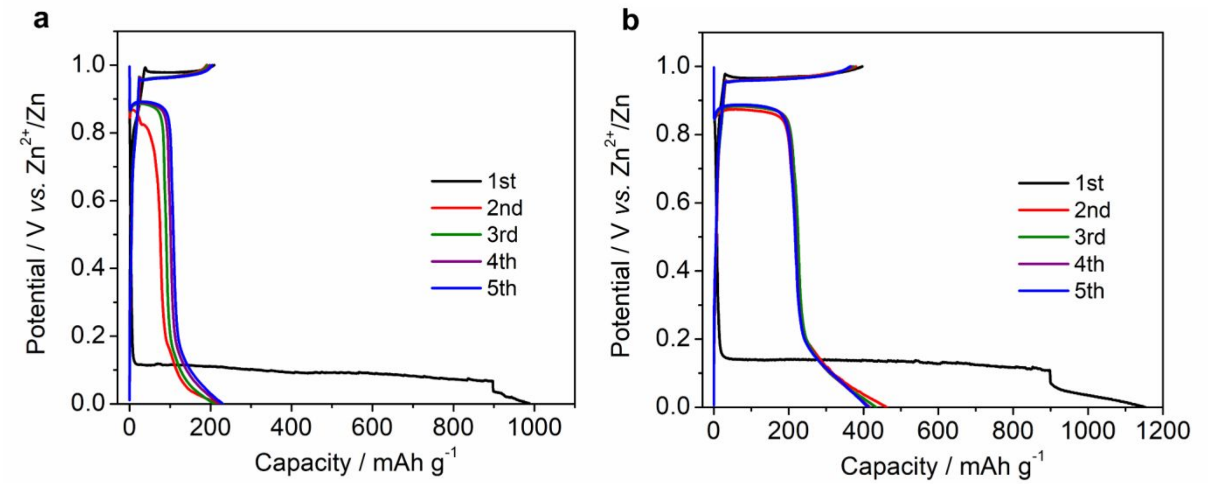

Figure S1. The GCD curves of the copper electrodes at 20 mA g^{-1}. (a) The micron-sized copper electrode; (b) The nano-sized copper electrode. As shown, there is a very large discharge capacity in the first cycle, which primarily results from the hydrogen evolution side reactions. This HER reaction promotes the formation of the zinc sulfate hydroxide precipitation.

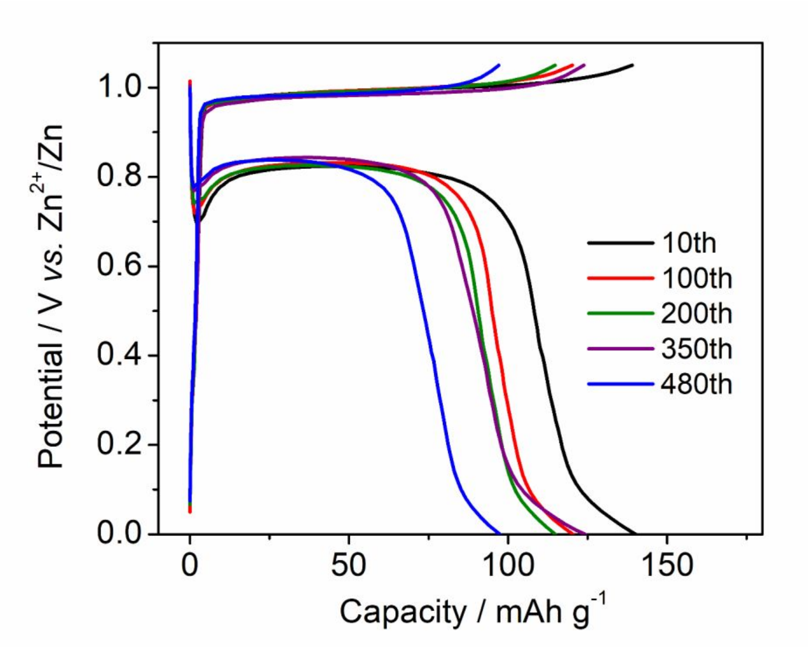

Figure S2. The selected GCD curves of the nano-Cu electrode at 200 mA g^{-1} during the cycling.

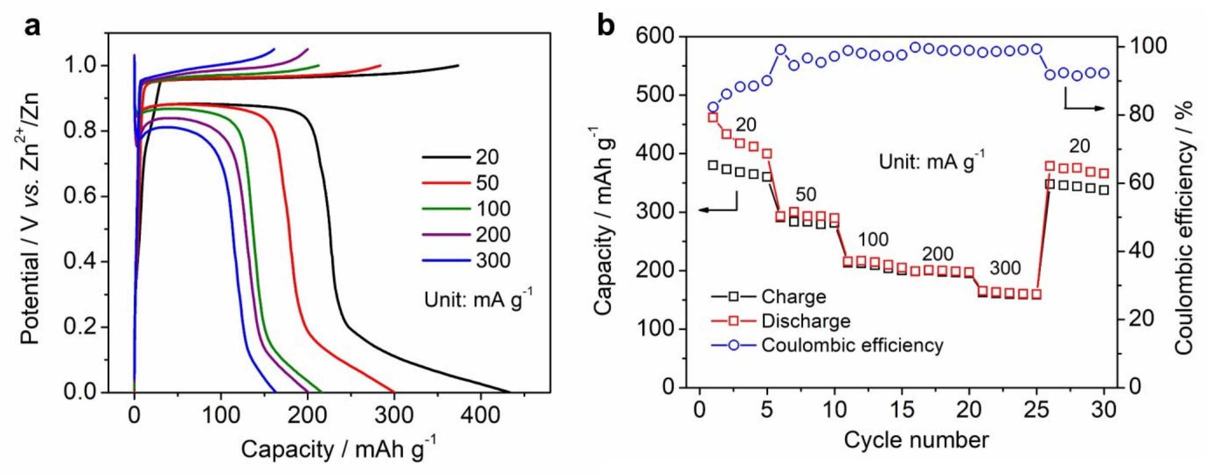

Figure S3. Rate performance of the rechargeable Voltaic batteries. (a) GCD curves at different current density; (b) Charge/discharge capacities and Coulombic efficiency in relation to the cycle number. To exclude the influence of the Zn metal anode polarization at higher current rates, we used 1.05 V as the cut-off potential for 200 and 300 mA g^{-1}.

Table S1. The cycling comparison of representative Zn-Cu Voltaic batteries.

Reference	Electrolytes	Separators	Capacity	Capacity retention	Voltage	Notes
[1]	2 M $LiNO_3$ for Cu, and 1 M $Zn(NO_3)_2$ for Zn	$Li_{1+x+y}Al_xTi_{2-x}Si_yP_{3-y}O_{12}$ solid-state electrolyte (SSE) film	843 mAh g^{-1}	~100% after 150 cycles	~0.8 V	(1) Expensive SSE; (2) Increased mass burden due to the $LiNO_3$ salt; (3) Large polarization (~0.5 V).
[2]	$ZnSO_4$ + $CuSO_4$ + Na_2SO_4 background electrolyte	Neosepta CIMS monovalent cation exchange membrane	763 mAh g^{-1}	~100% after 100 cycles	~0.7 V	(1) Complicated cell design; (2) Large polarization of ~0.6 V is observed.
[3]	$ZnSO_4$ + $CuSO_4$ electrolytes	Na-ion hydrogel	280 mAh g^{-1}	~43% after 100 cycles	~0.7 V	Poor cycling stablility.
[4]	1 M KOH aqueous electrolyte	NKK separator	718 mAh g^{-1}	~50% after 200 cycles	~0.75 V	Zn anode corrosion, and poor cycling stability
[5]	45 wt.% KOH aqueous electrolyte	Cellulose and Celgard separator	550 mAh g^{-1}	~55% after 200 cycles	~0.80 V	Zn anode corrosion, and poor cycling stability
[6]	Aqueous $ZnCl_2$ plus non-aqueous [bmmim]TFSI ionic liquids	Cation or anion exchange membrane	395 mAh g^{-1}	~80% after 2000 cycles	~1.0 V	(1) This ionic liquid is highly expensive and acute toxic; (2) Ion exchange membranes are expensive
**This work**	1 M $ZnSO_4$	Glass fiber papers	370 mAh g^{-1}	~71% after 485 cycles	~0.85 V	(1) Simple and cheap cell components; (2) Compitable with current battery manufactruing.

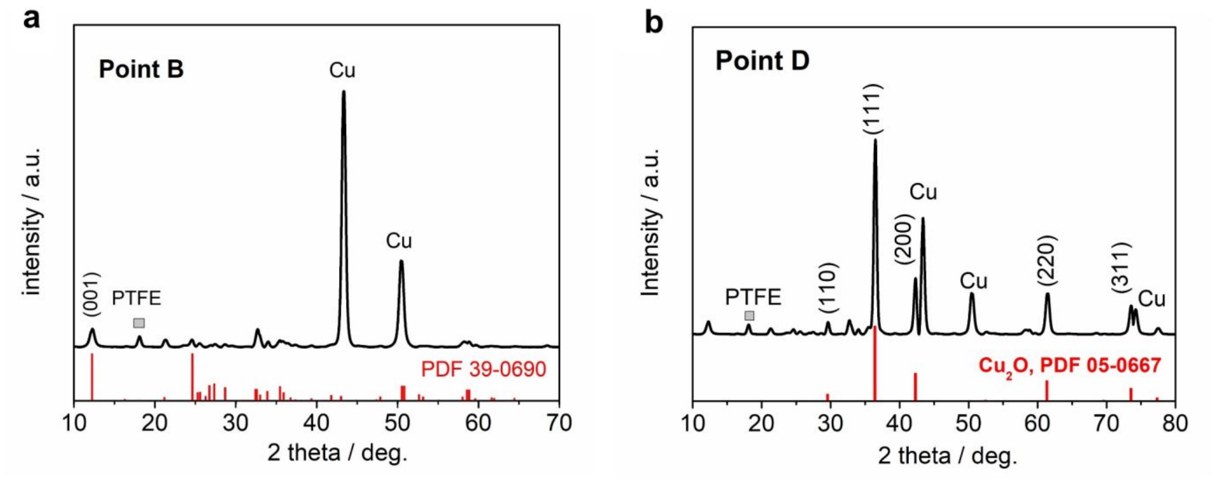

Figure S4. The index of XRD results to the standard diffraction patterns. (a) The XRD result of the electrode at point B (fully discharged); (b) The XRD result of the electrode at point D (fully charged). As shown, the fully discharged electrode exhibits multiple peaks at 12.3, 21.4, 24.6, 27.6, 32.7, and 34.1°, which can be well ascribed to the $Zn_4SO_4(OH)_6{\cdot}H_2O$ phase (PDF 39-0690). For the fully charged electrode, there are new peaks showing up at 29.5, 36.3, 42.2, 61.4, and 73.5°, which are indexed to the Cu_2O material (PDF 05-0667).

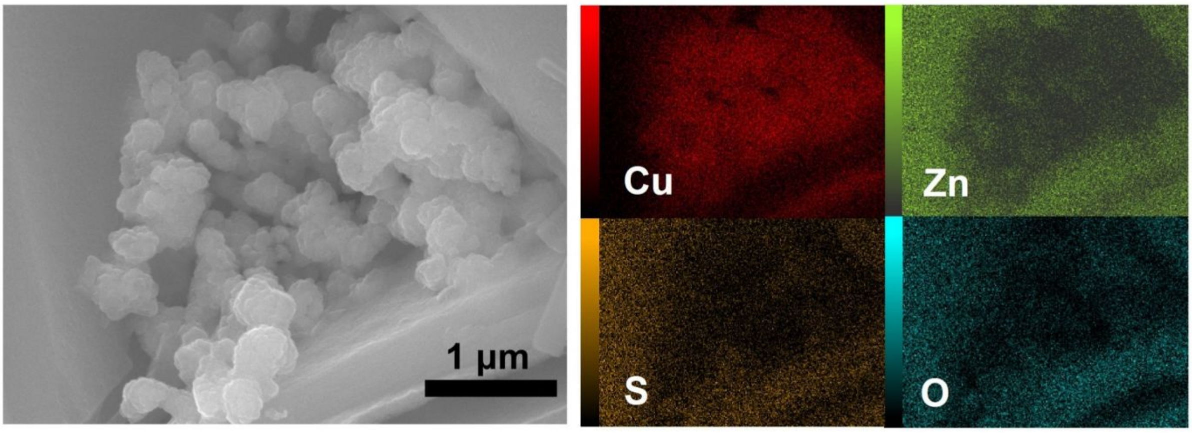

Figure S5. The SEM image and the elemental mapping of the nano-Cu electrode at the point B stage. As shown, the nano-sized Cu is well embedded and surrounded by the $Zn_4SO_4(OH)_6 \cdot H_2O$ precipitation, which facilitates the subsequent Cu/Cu_2O conversion.

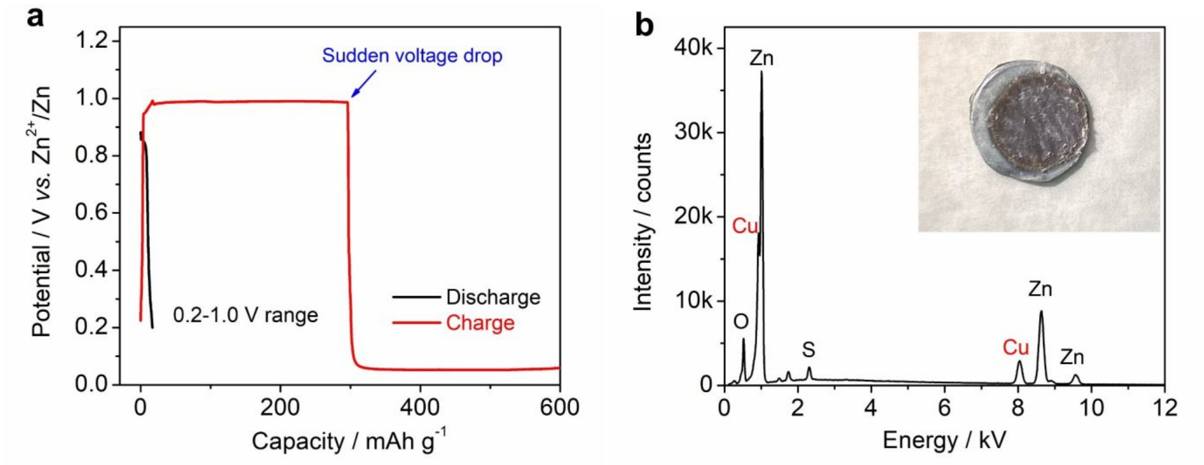

Figure S6. (a) The GCD curves of the nano-Cu electrode at 20 mA g^{-1} in the 0.2-1.0 V range; (b) The picture of the Zn metal anode and the EDS analysis. As shown, if we limit the discharge voltage to 0.2 V, the discharge capacity is minimal, and the nano-Cu electrode will not form the $Zn_4SO_4(OH)_6$ precipitation. When the nano-Cu electrode starts charging, there is a sudden potential drop after the capacity reaches 300 mAh g^{-1}. We disassembled the battery, and we found red deposits on the Zn metal anode. EDS reveals that the Cu element appears on the Zn metal anode, indicating the Cu^{2+} dissolution and crossover issue when $Zn_4SO_4(OH)_6$ is absent.

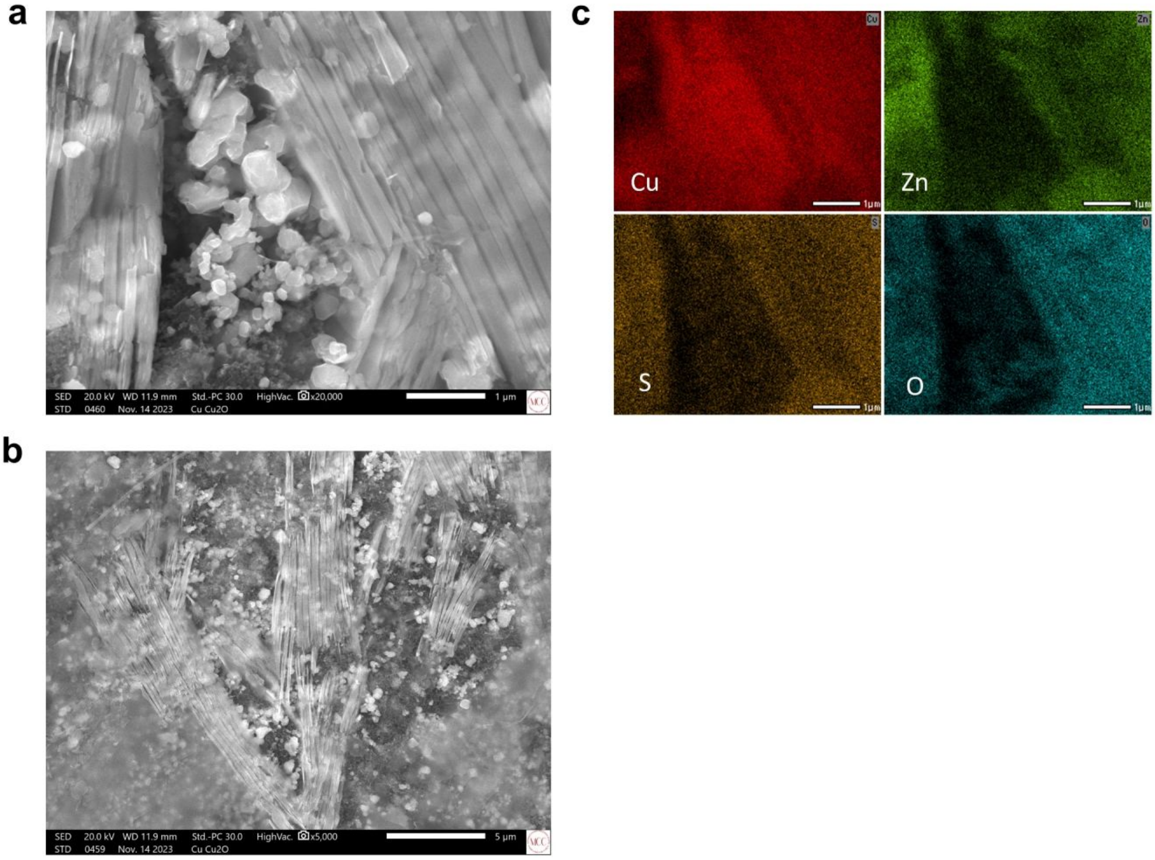

Figure S7. (a-b) SEM images of the fully charged nano-Cu electrode; (c) The EDS mapping result. As shown, the Cu electrode is well dispersed in the $Zn_4SO_4(OH)_6$ matrix, which benefits the Cu/Cu_2O conversion reaction.

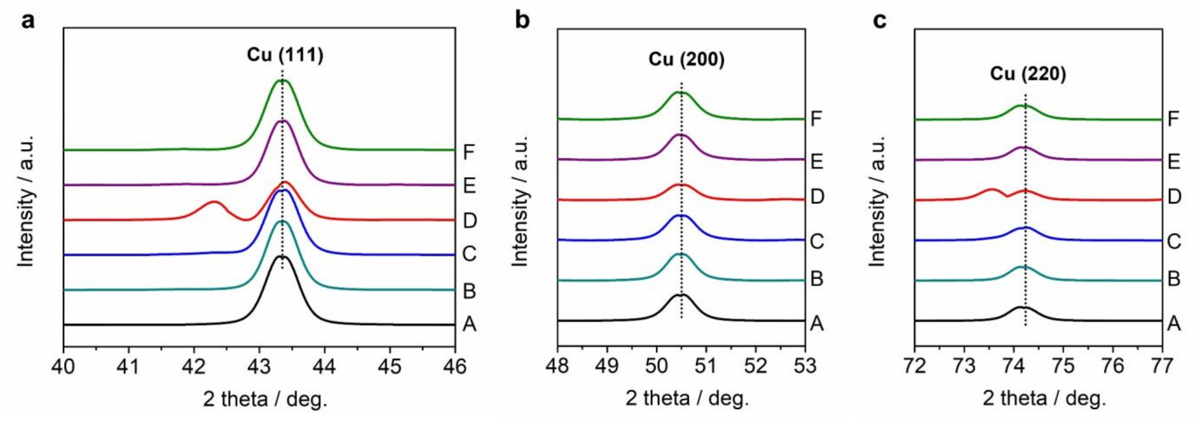

Figure S8. The zoom-in images of the ex-situ XRD patterns. (a) XRD results from 40 to 46 degrees; (b) XRD results from 48 to 53 degrees; (c) XRD results from 72 to 77 degrees. As shown, there is no noticeable peak shift for the nano-Cu electrode during the entire charge/discharge process, which rules out the $Zn_{0.18}Cu$ alloy formation.

Reference:

[1] X. Dong, Y. Wang, Y. Xia, *Sci. Rep.* **2014**, *4*, 1-6.

[2] A. Jameson, A. Khazaeli, D. P. J. Barz, *J. Power Sources* **2020**, *453*, 227873.

[3] S. Mypati, A. Khazaeli, D. P. J. Barz, *J. Energy Storage* **2021**, *42*, 103109.

[4] Q. Zhu, M. Cheng, B. Zhang, K. Jin, S. Chen, Z. Ren, Y. Yu, *Adv. Funct. Mater.* **2019**, *29*, 1905979.

[5] D. J. Arnot, N. B. Schorr, I. V. Kolesnichenko, T. N. Lambert, *J. Power Sources* **2022**, *529*, 231168.

[6] C. Xu, C. Lei, J. Li, X. He, P. Jiang, H. Wang, T. Liu, X. Liang, *Nat. Commun.* **2023**, *14*, 2349.
